# DNA-Dependent Protein Kinase Inhibitor Peposertib Potentiates the Cytotoxicity of Topoisomerase II Inhibitors in Synovial Sarcoma Models

**DOI:** 10.3390/cancers16010189

**Published:** 2023-12-30

**Authors:** Steffie Revia, Magdalena A. Budzinska, Olga Bogatyrova, Felix Neumann, Astrid Zimmermann, Christiane Amendt, Joachim Albers

**Affiliations:** 1 Research Unit Oncology, Merck Healthcare KGaA, Frankfurter Str. 250, 64293 Darmstadt, Germany; steffie.revia@merckgroup.com (S.R.);; 2 Ardigen S.A., Podole 76, 30-394 Cracow, Poland

**Keywords:** synovial sarcoma, DNA repair, NHEJ, DNA-PK, peposertib

## Abstract

**Simple Summary:**

This study focuses on finding improved treatment strategies for advanced or metastatic synovial sarcoma, which is a rare and aggressive type of soft tissue sarcoma. The researchers tested a combination of two drugs, peposertib and doxorubicin, to see if they could work together to kill synovial sarcoma cells more efficiently. The experiments conducted in cultured cancer cells and mouse models of synovial sarcoma demonstrated that when these drugs were used together, they had a significantly stronger effect against cancer cells compared to using either drug alone. It is noteworthy that the combination could successfully overcome resistance to doxorubicin monotherapy in a patient-derived tumor model. This study also shed light on the underlying molecular mechanisms of this combination effect. Overall, the findings suggest that combining peposertib with doxorubicin could be a promising treatment option for synovial sarcoma patients in the future.

**Abstract:**

Synovial sarcoma is a rare and highly aggressive subtype of soft tissue sarcoma. The clinical challenge posed by advanced or metastatic synovial sarcoma, marked by limited treatment options and suboptimal outcomes, necessitates innovative approaches. The topoisomerase II (Topo II) inhibitor doxorubicin has remained the cornerstone systemic treatment for decades, and there is pressing need for improved therapeutic strategies for these patients. This study highlights the potential to enhance the cytotoxic effects of doxorubicin within well-characterized synovial sarcoma cell lines using the potent and selective DNA-PK inhibitor, peposertib. In vitro investigations unveil a p53-mediated synergistic anti-tumor effect when combining doxorubicin with peposertib. The in vitro findings were substantiated by pronounced anti-tumor effects in mice bearing subcutaneously implanted tumors. A well-tolerated regimen for the combined application was established using both pegylated liposomal doxorubicin (PLD) and unmodified doxorubicin. Notably, the combination of PLD and peposertib displayed enhanced anti-tumor efficacy compared to unmodified doxorubicin at equivalent doses, suggesting an improved therapeutic window—a critical consideration for clinical translation. Efficacy studies in two patient-derived xenograft models of synovial sarcoma, accurately reflecting human metastatic disease, further validate the potential of this combined therapy. These findings align with previous evidence showcasing the synergy between DNA-PK inhibition and Topo II inhibitors in diverse tumor models, including breast and ovarian cancers. Our study extends the potential utility of combined therapy to synovial sarcoma.

## 1. Introduction

Synovial sarcoma (SS) is a rare but highly malignant type of soft tissue sarcoma (STS), and accounts for 5–10% of all STS [1,2]. In >95% of cases, SS is associated with a chromosomal translocation involving genes encoding the SWI-SNF complex component SS18 (formerly SYT) and a SSX transcriptional repressor. The translocation results in the formation of an in-frame fusion involving SS18 with SSX1, SSX2 and, less frequently, SSX4. This fusion event gives rise to SS18-SSX proteins, which play a pivotal role in driving the process of sarcomagenesis and hold essential diagnostic significance [3,4,5,6].

The current recommended approach for treating localized synovial sarcoma continues to be surgical removal of the tumor with clear margins, supplemented by radiotherapy and/or chemotherapy, which are determined based on individual patient and tumor characteristics. [1,7]. More than 50% of SS patients develop metastatic disease, which is treated mostly with anthracycline-based chemotherapy regimens and palliative intent [2,8]. Doxorubicin in monotherapy, or in combination with ifosfamide, is considered as the SOC in first-line with average response rates of ~20% and ~30%, respectively, although this is not based on prospective randomized SS specific studies due to the rarity of the disease and the fact that STS have been clinically treated as one type of disease for a long time [2,5,9].

Anthracyclines such as doxorubicin exert their cytotoxic effects by inhibiting type II topoisomerase (Topo II) enzymes, which are essential for DNA replication and transcription [10]. The mechanism of action of Topo II inhibitors involves the stabilization of the cleavage complex formed by Topo II and DNA, preventing the enzyme from re-ligating the break and releasing the DNA [11]. The stabilized cleavage complex then becomes a cytotoxic intermediate, ultimately leading to DNA double strand breaks (DSB) [12]. In mammalian cells, the nonhomologous-end-joining (NHEJ) repair pathway is critical for repairing Topo II-mediated DNA damage [11,13]. DNA-dependent protein kinase (DNA-PK) is an enzyme that belongs to the serine/threonine kinase family, and plays a crucial role in NHEJ mediated repair. It works in collaboration with five other factors, namely Ku70, Ku80, XRCC4, ligase IV, and Artemis [13,14]. The identification of DNA-PK’s crucial role in DNA damage repair has brought attention to the potential use of DNA-PK inhibitors to impede the repair process and increase the effectiveness of DNA-damaging agents [14,15]. Peposertib is an orally bioavailable inhibitor of DNA-PK, exhibiting strong potency and selectivity. In preclinical models, it has been shown to significantly enhance the antitumor effects of ionizing radiation and DNA double-strand break-inducing agents, including anthracyclines like doxorubicin [14]. In the clinic, peposertib is currently being evaluated in combination with various DNA damaging agents, including external beam radiotherapy (NCT04555577; NCT04533750), radiopharmaceuticals (NCT05868174; NCT04750954; NCT04071236), chemotherapies (NCT03983824; NCT04092270; NCT05711615), and the ATR inhibitor tuvusertib (NCT05687136).

Consequently, we put forth the hypothesis that peposertib in conjunction with Topo II inhibitors will yield superior outcomes compared to individual agent treatments in synovial sarcoma models. In this study, we present compelling preclinical evidence of the synergistic antitumor effects achieved by combining peposertib with Topo II inhibitors, with a particular emphasis on doxorubicin, in human synovial sarcoma tumor models.

## 2. Materials and Methods

### 2.1. Reagents and Cell Culture

Peposertib was synthesized at Merck Healthcare KGaA, Darmstadt, Germany. Doxorubicin and Etoposide were purchased from Sigma-Aldrich. For in vitro experiments, drugs were solubilized in DMSO to create stock solutions, which were then frozen and stored at −20 °C until needed. The concentration of DMSO in the media did not exceed 0.1% (*v/v*).

SYO-1 cells were kindly provided by Okayama University, Okayama City, Japan, and the HS-SY-II cell line was purchased from RIKEN cell bank. Both cell lines were cultured in Dulbecco’s Modified Eagle Medium (DMEM) + 10% fetal calf serum at 37 °C with 10% CO_2_. Cell line identity was confirmed by short tandem repeats (STR) analyses, mycoplasma and bacterial contamination was excluded. Expression of the pathognomonic SS18-SSX fusion gene in both cell lines was confirmed by Western blotting.

### 2.2. Cell Viability Assay

For viability and combination matrix assays, SYO-1 and HS-SY-II cells were plated at 3000 and 7500 cells per well, respectively, in 96-well plates. The next day, cells were treated with serial dilution of drugs using a Tecan D300e Digital Despenser, and DMSO concentration was normalized in all wells. At 168 h (7 days) following the drug treatment, the effect on cell growth or viability was assessed with Resazurin assay according to the manufacturer’s protocol and a fluorescent signal was recorded (560Ex/590Em) using a Tecan Infinite M200 plate reader.

Dose response curve and IC_50_ values were generated using Graphpad Prism (v9.0.0), while for the combination matrix, synergism was analyzed with Loewe’s additivity model using GeneData Screener Software (Version 19.0.5) and graphed using Combenefit software (v.2.021) [16].

### 2.3. Immunoblotting

Cells were harvested and lysed in RIPA buffer: 20 mM Tris-HCl (pH 7.5), 150 mM NaCl, 1 mM Na2EDTA, 1 mM EGTA, 1% NP-40, 1% sodium deoxycholate, 2.5 mM sodium pyrophosphate, 1 mM beta-glycerophosphate, 1 mM Na3VO4, 1 µg/mL leupeptin (Cell Signaling Technology, Danvers, MA, USA) supplemented with both protease and phosphatase inhibitors (Roche Diagnostics, Rotkreuz, Switzerland). To ensure lysis, cells were sonicated with Diagenode Bioruptor Plus for 10 min (30 s on/30 s off cycle) at 4 °C and subsequently centrifuged at 4 °C at 13,000 rpm. Protein concentration was determined by the BCA protein assay (Thermo Fisher Scientific, Waltham, MA, USA) and an equal amount of protein was mixed with 4x NuPAGE LDS Sample Buffer (Invitrogen, Waltham, MA, USA) and 10× NuPAGE Reducing Agent (Invitrogen). Samples were heated at 70 °C for 10 min prior separation on NuPAGE 4–12% BisTris Mini Protein gels (Invitrogen) and NuPAGE MOPS SDS Running Buffer (Invitrogen). Proteins were subsequently transferred to polyvinylidene fluoride (PVDF) membrane via iBlot Dry Blotting System (Thermo Fisher Scientific, Waltham, MA, USA). Membranes were incubated with the appropriate antibodies and imaged with Bio-rad ChemiDoc Imaging System using Western Lighting Plus ECL (PerkinElmer, Waltham, MA, USA). The list of antibodies and their sources can be found in Supplementary Table S2.

### 2.4. IncuCyte Live Cell Imaging 

SYO-1 and HS-SY-II cells were plated in 96-well plates and incubated overnight before drugs and IncuCyte Annexin V red reagent (Essen Bioscience, Ann Arbor, MI, USA) was added the next day to label apoptotic cells in real time. Cells were imaged using 10× objective in Incucyte S3 device at 2 h intervals for 7 days. Relative apoptosis events were determined by the number of Annexin V red counts per mm^2^ normalized to percent confluence.

### 2.5. RNA Extraction, NanoString nCounter Assay and the Analysis

Total RNA was isolated using RNAqueous™-4PCR Total RNA Isolation Kit (Invitrogen) according to the manufacturer’s protocol and its concentration was measured with a Qubit 4.0 fluorometer. For the gene expression analysis, 100 ng of isolated total RNA were assessed using the nCounter PanCancer Pathway Panel (NanoString, Seattle, WA, USA) according to the manufacturer’s instruction. 

Data analysis and processing were conducted by integrating three independent quantification methods: DESeq2, Limma, and nSolver, with corresponding R packages (DESeq2 and limma). Background correction was performed by subtracting the value of “mean + 2× standard deviation” obtained from the negative controls from the raw counts. Further, adjusted raw counts were normalized to the geometric mean of six positive controls in each sample, followed by normalization using the geometric mean of 40 internal reference genes. Data exclusion criterion was set at a minimum of 30 counts of mRNA template. For multiple testing correction, the Bonferroni-Hochberg method was utilized, applying an adjusted *p*-value threshold of *p* = 0.05. Moreover, a log2-fold change (log2FC) threshold of 1 was selected as the criterion for identifying differentially regulated genes. A gene was classified as differentially expressed if it met the threshold criteria in at least two out of three quantification methods.

Pathway enrichment analysis was performed using Fisher’s exact test with a significant threshold of *p*-value = 0.05, since nCounter PanCancer Pathway Panel comes with 770 genes. 

### 2.6. Animal Studies 

In vivo efficacy data were generated in subcutaneous human cell-line-derived xenograft and patient-derived xenograft (PDX) models. For human cell-line-derived xenograft tumors, 5 million SYO-1 cells were injected subcutaneously (s.c) in 1:1 (*v:v*) DPBS/Matrigel Basement Membrane Matrix into the right flanks of female 8–10 weeks old H2d Rag2 [C;129P2-H2^d^-TgH(II2rg)^tm1Brn^-TgH(Rag2)^tm1Alt^N4] mice (Taconic Biosciences). The study was randomized into groups (N = 10/group) of equal mean tumor volume (TV) prior to treatment. All studies were approved by the local animal welfare authority (Regierungspräsidium Darmstadt, Hesse, Germany; experimental license number DA4/Anz.1040).

The efficacy studies in the synovial sarcoma PDX models CTG-1173 and CTG-2004 were performed at Champions Oncology according to the guidelines of the Institutional Animal Care and Use Committee (IACUC) of Champions Oncology. For these PDX studies, stock mice were bilaterally implanted with fragments from each of the 2 Champions TumorGraft^®^ models CTG-1173, CTG-2004. After tumors reached 1000–1500 mm^3^, they were harvested, and tumor fragments were implanted s.c. in the left flank of female study mice. Tumor growth was monitored twice a week using digital calipers, and TV was calculated using the formula (0.52 × [length × width^2^]). When TV reached approximately 150–300 mm^3^, animals were matched by tumor size and assigned into vehicle control or treatment groups (n = 8/group), and dosing was initiated on d0 up to d52 (CTG-1173) or up to d34 (CTG-2004) or until mean TV in one group reached 1500 mm^3^. Tumor size and body weight were measured twice a week. Histopathological and molecular analyses were performed at Champions Oncology, and data was reviewed at Merck Healthcare KGaA.

For all in vivo studies, peposertib was formulated in vehicle (0.5% Methocel, 0.25% Tween20, 300 mmol/L sodium citrate buffer, pH 2.5 and administered orally. Doxorubicin or pegylated liposomal doxorubicin formulated for intravenous administration in 5% (50 mg/mL) glucose solution was injected into the tail vein once weekly at the indicated dose.

## 3. Results

### 3.1. Peposertib Enhances the Cytotoxicity of Topo II Inhibitors in Synovial Sarcoma Cell Lines

**Figure 1:**
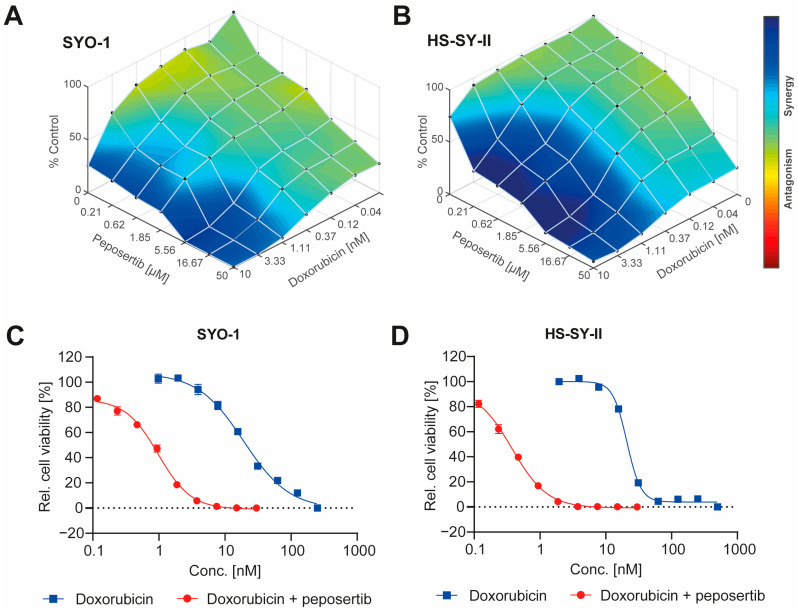
Peposertib synergistically enhances the cytotoxicity of doxorubicin in synovial sarcoma cells. Overlay of Loewe synergy scores (**A**) SYO-1 and (**B**) HS-SY-II cells with combinations of doxorubicin and peposertib. Cell viability data were used to impute the synergy scores using Combenefit software. Potentiation of doxorubicin cytotoxicity by 1 µM peposertib on (**C**) SYO-1 and (**D**) HS-SY-II cells as measured using an Alamar Blue viability assay 168 h post treatment.

We selected two SS18:SSX fusion positive synovial sarcoma cell lines, SYO-1 and HS-SY-II, to evaluate if DNA-PK inhibition by peposertib could enhance the cytotoxic effects of Topo II inhibitors, such as doxorubicin or etoposide. Single agent treatment with either doxorubicin or etoposide revealed comparable cell-killing activity in both cell lines while peposertib had notable cytotoxicity (IC50 = 18µM in SYO-1 and 21µM in HS-SY-II) only at concentrations far above those previously reported to be achievable in a clinical setting [17] (Supplementary Figure S1A–C). Multi-dose combinatorial response matrices over various concentration ranges demonstrated that concurrent treatment of peposertib with either doxorubicin or etoposide substantially decreased the viability of both cell lines as assessed by an Alamar Blue viability assay. By systematically mapping the combination matrix data onto the Loewe model, the presence of synergistic effects (BLISS score >2.0) between peposertib and the two Topo II inhibitors was confirmed (Figure 1A,B and Supplementary Figure S1D,E). This synergy was particularly evident when considering the sublethal concentration ranges pertinent to individual monotherapy treatments. For a subsequent confirmatory study, we selected a sublethal peposertib concentration of 1µM co-administered with increasing concentrations of doxorubicin. This resulted in a pronounced reduction of the doxorubicin IC_50_ from 19 nM to 0.9 nM (22-fold) in SYO-1 and from 21 nM to 0.4 nM (52-fold) in HS-SY-II cells (Figure 1C,D). A comparable effect was observed when 1 µM of peposertib was combined with etoposide (Supplementary Figure S1F,G).

**Figure 2:**
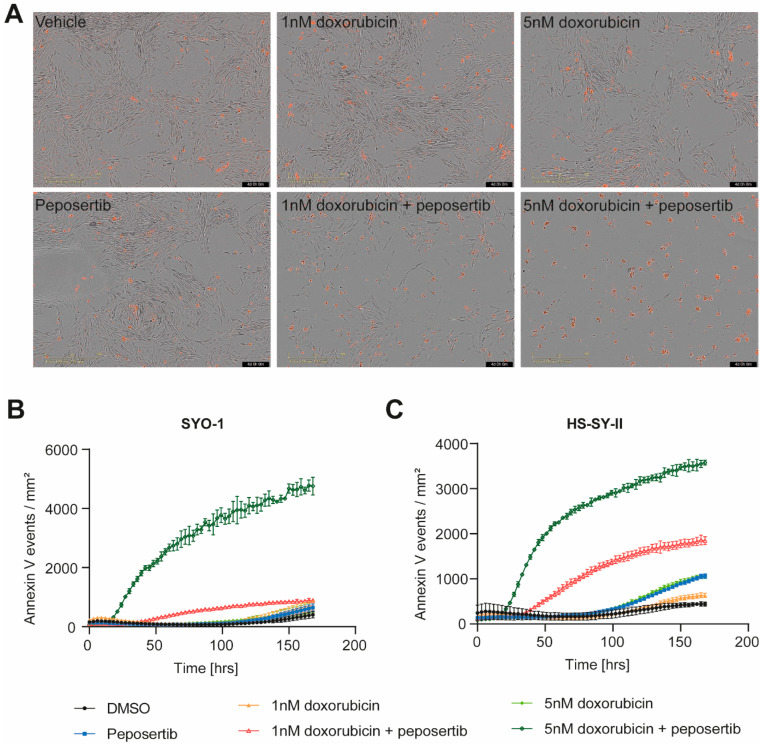
Combined treatment of peposertib and doxorubicin induces apoptotic cell death in synovial sarcoma cell lines. (**A**) Incucyte^®^ bright-field images of SYO-1 at 96 h post treatment at 10× magnification overlay with AnnexinV-Red staining. Images represent 3 independent replicates. Quantification of Annexin V-Red positive cells in (**B**) SYO-1 and (**C**) HS-SY-II over the course of treatment as monitored using the Incucyte^®^.

To corroborate the findings obtained from the Alamar Blue viability assay, we utilized Incucyte^®^ live cell imaging to monitor alterations in cell growth and death (apoptotic cells detected by Annexin V Red staining) throughout a 168 h duration. We focused on the combination treatment of 1μM peposertib with two concentrations of doxorubicin, 1 nM and 5 nM. In the scenario of the low-dose combination (1 nM doxorubicin with 1µM peposertib), a qualitative assessment of bright-field images overlayed with Annexin V Red staining unveiled a notable decrease in cell density at 72 or 96 h subsequent to co-administration of peposertib and doxorubicin, as compared to the individual treatments (Figure 2A, Supplementary Figure S2A,B). This observed decrease in cell density was accompanied by a minor elevation of Annexin V staining in SYO-1 cells and moderate increase in HS-SY-II cells (Figure 2B,C). In contrast, the co-administration of 5 nM doxorubicin and peposertib resulted in a pronounced augmentation of Annexin V-positive cells in both cell lines (Figure 2A and Supplementary Figure S2A). Quantitative analyses of the Incucyte^®^ bright-field images confirmed both the cytostatic and cytotoxic natures of the low-dose and high-dose treatment regimens (Figure 2B,C and Supplementary Figure S2B).

### 3.2. Peposertib Synergizes with Doxorubicin to Inactivate DNA Repair Pathways and Activate p53 Tumor Suppressor Genes Simultaneously

**Figure 3:**
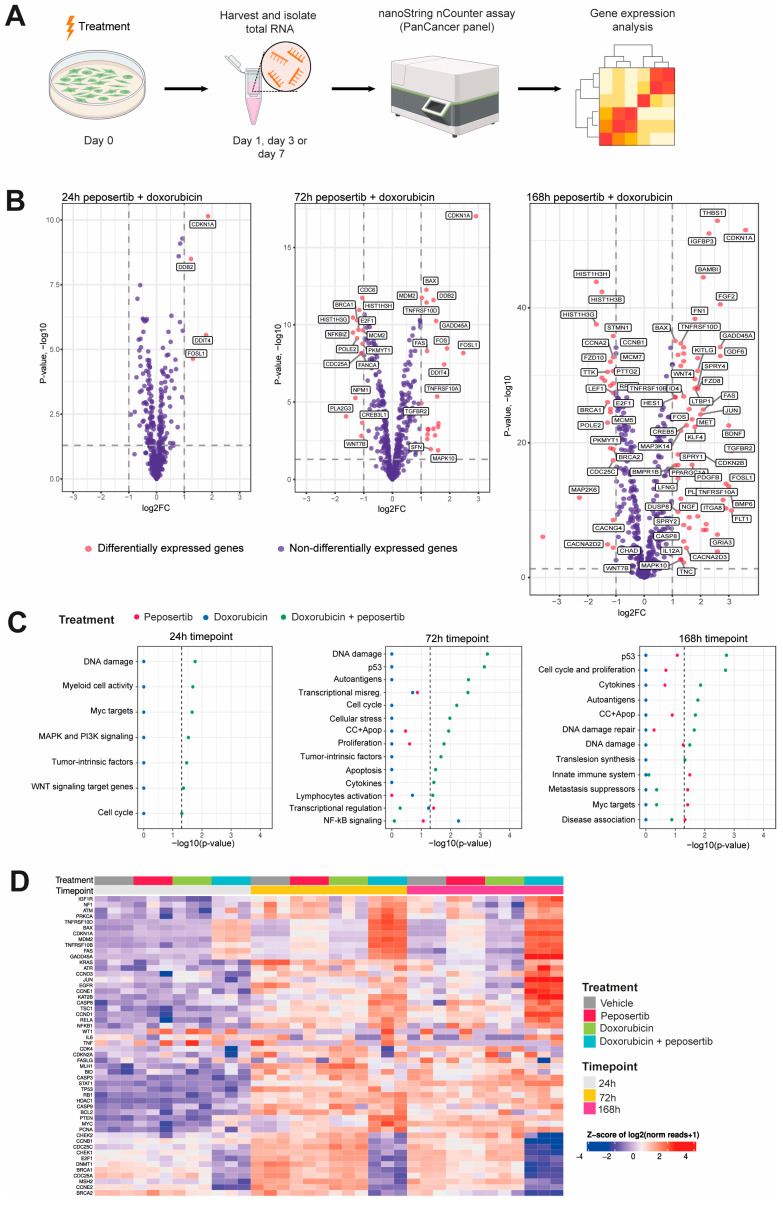
Gene expression analysis revealed induction of p53- and DNA damage repair signaling upon combined treatment with doxorubicin and peposertib. (**A**) Scheme of the experimental setup for gene expression analysis in SYO-1 cells using the NanoString nCounter^®^ PanCancer panel. (**B**) Volcano plots highlighting differentially regulated genes (red dots) identified by limma upon combination treatment for 24, 72 and 168 h. The dotted horizontal and vertical lines indicate the adjusted *p*-value of 0.05 and log2FC of 1, respectively, whereas the names indicate differentially regulated genes identified by at least two methods. (**C**) Pathway enrichment analysis at 24, 72 and 168 h. Significantly enriched pathways were identified using Fisher’s exact test. Dashed line indicates the enrichment significance of the *p*-value (0.05). DE differentially expressed genes; non-DE not differentially expressed genes. (**D**) Heatmap showing expression levels of selected p53 related genes included in the Nanostring nCounter panel. Each column represents one biological replicate.

In order to study the underlying molecular mechanism driving the observed synergistic effects, we opted to employ the NanoString nCounter^®^ PanCancer pathways panel, which allowed us to scrutinize transcriptomic alterations in 770 genes across 13 cancer-associated canonical intracellular signaling pathways. We collected total RNA from SYO-1 cells that had been treated with either single agent or combination treatment for 24, 72, and 168 h (Figure 3A). We used an adjusted *p*-value of 0.05 and log2FC of 1 as a threshold to define whether a gene was differentially regulated in response to treatment. Panel assessment of single-agent treatment with either doxorubicin or peposertib revealed that only very few genes exhibit differential regulation throughout the 168 h of treatment (Supplementary Figure S3A–C). By contrast, co-treatment with doxorubicin and peposertib consistently impacted a greater set of genes across the cancer-associated signaling pathways, particularly after 72 h and 168 h treatment (Figure 3B and Supplementary Figure S3A). To be precise, we observed that a total of 66 genes exhibited significant upregulation, whereas 30 genes displayed significant downregulation upon combined treatment at the 168 h timepoint. A subsequent pathway enrichment analysis revealed that the majority of genes that were differentially regulated in the combined treatment are associated with p53-signaling and DNA damage repair (Figure 3C). Heatmap visualization of p53-associated genes further revealed the time-dependent effects and synergistic impact of the combined treatment on the p53 pathway (Figure 3D).

### 3.3. Concurrent Administration of Peposertib and Doxorubicin Triggers p53 Mediated Apoptosis in Synovial Sarcoma Cell Lines

**Figure 4:**
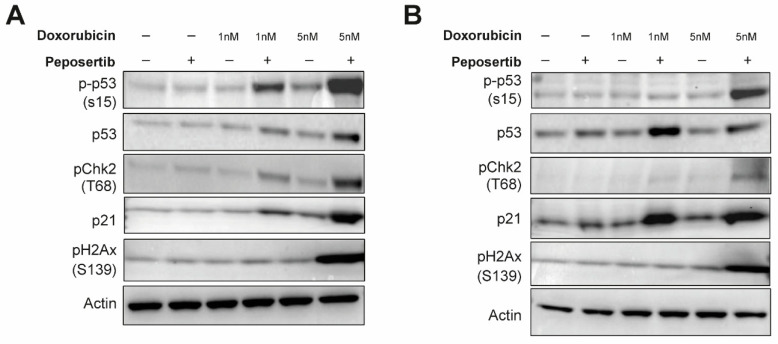
Peposertib in combination with doxorubicin activates p53 signaling and suppresses DNA repair. SYO-1 (**A**) and HS-SY-II (**B**) cells were exposed to doxorubicin, 1 μM peposertib or their combination for a period of 24 h. Protein lysates were analyzed by Western blotting using antibodies against phospho-Chk2 (T68), phospho-p53 (S15), p21, phospho-H2Ax (S139), and actin (loading control). Induction of p53 signaling pathway and accumulation of DNA-damage is observed in the combination groups with higher doxorubicin dose for both cell lines. The uncropped blots are shown in Figure S5

Taking into account the data from our gene expression analysis and the mechanisms of action of both drugs, we hypothesized that disruptions in DNA damage repair pathways and p53-mediated signaling constitute a central theme underlying the mode of action of the synergistic effects. To further investigate this, we collected whole cell lysates from SYO-1 and HS-SY-II cells treated with single-agents or combinations for 24 h and immunoblotted selected markers of DNA damage and repair signaling. Using γ-H2A.x as a generic proxy for DNA damage, it was observed that the combination of low-dose doxorubicin (1 nM) and peposertib did not induce levels of DNA damage different from the control treatments (Figure 4A,B). When a higher concentration of 5 nM doxorubicin was combined with peposertib, the evident occurrence of apoptosis described earlier in the manuscript is accompanied by a noticeable increase in γ-H2A.x, an indicator for accumulation of unrepaired DNA damage (Figure 4A,B). Elevated levels of phosphorylated p53 (Ser15) were detected in both cell lines, concomitant with an increase in protein expression of the cyclin-dependent kinase inhibitor p21, a transcriptional target of p53 (Figure 4A,B). This finding is consistent with the gene expression analysis data (Figure 3), which demonstrated strong upregulation of p53-dependent genes including CDKN1A, the gene encoding p21. Additionally, our Western blot analysis showed that Checkpoint kinase 2 (Chk2), a crucial mediator of various cellular responses to genotoxic stress, was phosphorylated (Thr68) upon treatment with the combination therapy.

### 3.4. Peposertib Enhances the Anti-Tumor Activity of Doxorubicin In Vivo

**Figure 5:**
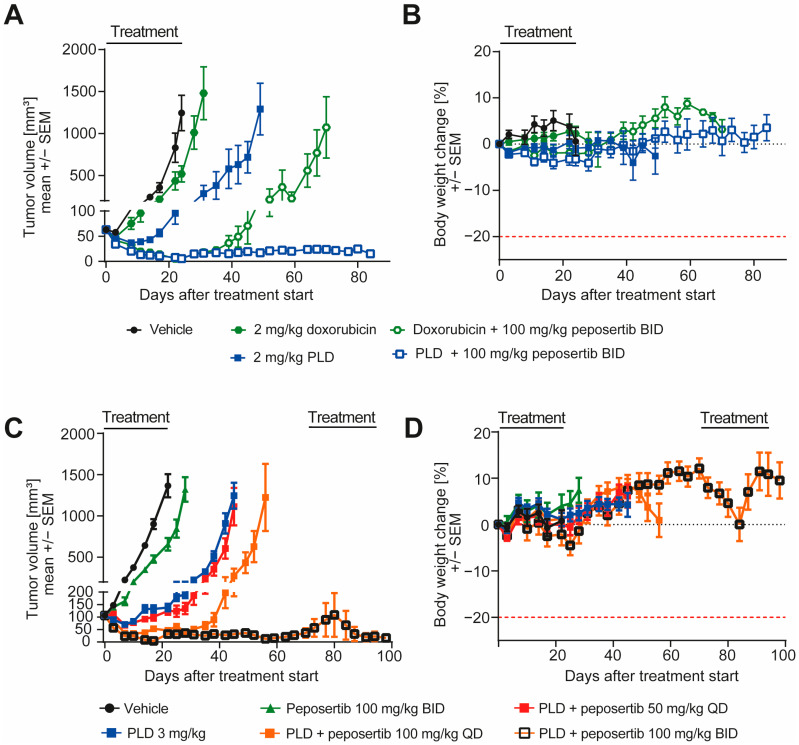
Peposertib enhances the anti-tumor activity of doxorubicin in subcutaneous SYO-1 xenograft tumors. (**A**,**C**): tumor growth of SYO-1 xenografts treated with vehicle, peposertib, doxorubicin, PLD or combinations of doxorubicin or PLD with peposertib (n = 10 for all groups, mean ± SEM). (**B**,**D**): relative body weight changes of SYO-1 tumor-bearing mice throughout the studies. Statistical analysis was performed using two-way ANOVA. In (**A**), comparison of 2 mg/kg doxorubicin and 2 mg/kg PLD treatment at day 31 reached statistical significance, *p* < 0.0001. Comparison of the 2 combination arms also revealed statistically significant differences at day 71, *p* < 0.0001. For (**B**), anti-tumor activity of both PLD + peposertib 100 mg/kg QD and PLD + 100 mg/kg BID was statistically significant compared to PLD monotherapy, *p* < 0.001.

Next, we went on to investigate if peposertib is able to enhance the anti-tumor efficacy of doxorubicin in vivo in human xenograft models. In the clinic, doxorubicin can be administered in its pure, unmodified state, or as a pegylated liposomal formulation. The pegylated liposomal doxorubicin (PLD) formulation leverages advanced encapsulation techniques to optimize drug delivery and appears to have a favorable toxicity profile with better cardiac safety and less myelosuppression in the clinic [18]. We first compared the anti-tumor activity and tolerability of conventional doxorubicin with PLD in monotherapy and in combination with peposertib in mice harboring established subcutaneous SYO-1 xenografts. As monotherapies, doxorubicin or PLD were intravenously administered once weekly at equivalent doses of 2 mg/kg. For the dual therapy approach, peposertib was given orally two times a day (BID) with an 8 h interval between the two administrations, spanning across 4 days. This regimen commenced 24 h following the intravenous delivery of doxorubicin or PLD. A scheme of a weekly treatment cycle is provided in Supplementary Figure S4A. In this initial experiment, mice received four cycles of treatment. While doxorubicin monotherapy resulted in a moderate but statistically significant (*p* = 0.003) delay of tumor growth, treatment with PLD had a statistically significant superior anti-tumor efficacy (*p* < 0.001), and resulted in tumor regressions during the initial phase of treatment (Figure 5A). The addition of peposertib to conventional doxorubicin treatment significantly enhanced the anti-tumor activity and resulted in tumor regression during the course of the 4 weeks of treatment, but tumors started to regrow approximately 2 weeks after treatment stopped (Figure 5A). In contrast, when peposertib was combined with PLD treatment, durable anti-tumor responses were observed, and most tumors were still in regression even 60 days after the treatment was stopped (Figure 5A). Importantly, all administered treatments exhibited excellent tolerance, as indicated by clinical symptoms and body weight alterations in the treated animals (Figure 5B). This outcome not only reaffirmed the synergistic anti-tumor effects of doxorubicin and peposertib observed in vitro, but also highlighted the potential for an improved therapeutic window with the combination of peposertib and PLD, as evidenced in this tumor model. In a subsequent study, we investigated the dose-dependency of the anti-tumor effect of peposertib in combination with PLD. Mice harboring established subcutaneous SYO-1 xenografts were treated with once-weekly PLD, followed by 4 days treatment with either 50 mg/kg peposertib once daily (QD), 100 mg/kg QD, or 100 mg/kg BID. Peposertib monotherapy at the highest dose of 100 mg/kg BID administered for 4 days/week and PLD monotherapy were included as control treatments in this experiment. The animals enrolled in this study underwent randomization across the various treatment groups with a higher mean tumor volume than in the previous study to facilitate a more effective comparison of tumor regression across the different dose groups. Consistent with the results from our in vitro investigations, we did not detect substantial anti-tumor effects from peposertib monotherapy, and the anti-tumor activity of PLD was in line with our previous study (Figure 5C). The combination of PLD and 50 mg/kg QD demonstrated only marginal superiority over PLD monotherapy, but the addition of 100 mg/kg QD peposertib significantly enhanced the anti-tumor activity compared to PLD monotherapy (*p* < 0.0001, Figure 5C and Supplementary Figure S4B). The strongest anti-tumor activity was observed with co-treatment of PLD and 100 mg/kg BID peposertib, resulting in long-lasting tumor regression for up to 100 days post treatment start (Figure 5C) It is noteworthy that two tumors in this group relapsed approximately 40 days after cessation of treatment and began to undergo rapid regrowth. Upon a re-challenge with the same treatment regimen, both tumors promptly regressed again, indicating that tumor cells did not acquire resistance to the therapy during the initial treatment period (Supplementary Figure S4C). 

**Figure 6:**
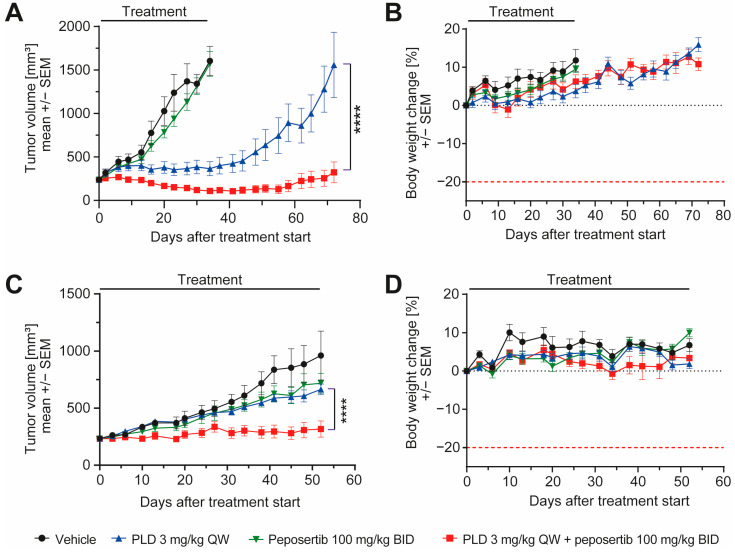
Peposertib enhances the anti-tumor activity of doxorubicin in synovial sarcoma PDX models. Tumor growth of (**A**) CTG-2004 and (**C**) CTG-1173 PDX treated with vehicle, peposertib, PLD or a combination (n = 8 for all groups, mean ± SEM). (**B**,**D**): corresponding mouse bodyweight change over time. The *p* values were calculated by two-way ANOVA, **** *p* < 0.0001.

To enhance the clinical relevance of our findings, we opted for two patient-derived synovial sarcoma xenograft models and conducted efficacy studies. The model CTG-1173 was derived from a lung metastasis of a 24-year-old female patient diagnosed with stage IV synovial sarcoma. Prior to acquisition of tissue for the establishment of the PDX model, the patient underwent two rounds of chemotherapy, involving a regimen containing doxorubicin. However, the patient exhibited no response to this treatment. The second model, CTG-2004, originated from a lung metastasis of a 30-year-old female patient diagnosed with stage IV synovial sarcoma. Molecular analysis carried out at Champions Oncology unveiled the existence of a SS18-SSX1 fusion in the CTG-1173 tumors, and the presence of SS18-SSX1 and SS18-SSX4 fusions in the CTG-2004 tumors, thus solidifying the categorization of the tumor type as synovial sarcoma. It is important to highlight that both tumors exhibited TP53 wildtype status, and lacked significant copy number alterations in any of the tested oncogenes. For a more comprehensive characterization, additional clinical, histological, and molecular data of the models can be found in Supplementary Table S1. For the efficacy study, female mice harboring subcutaneously implanted tumors were randomized into four groups and treated with 6 cycles of the treatment scheme that was applied in the previous studies (Supplementary Figure S4A). In CTG-2004, peposertib monotherapy had no effect on tumor growth compared to vehicle treatment, but PLD monotherapy significantly delayed tumor growth during the treatment period (*p* < 0.0001). Upon cessation of treatment, tumors started to regrow rapidly. In contrast, the combination treatment resulted in statistically superior (*p* < 0.0001) anti-tumor efficacy and tumor shrinkage during the treatment period (Figure 6A). As in the previous studies, all administered treatments exhibited excellent tolerance, as indicated by clinical symptoms and body weight (Figure 6B). In CTG-1173 xenografts, neither peposertib nor PLD in monotherapy significantly inhibited tumor growth (Figure 6C). The resistance to PLD exhibited by these tumors aligns with the lack of responsiveness observed in the patient, who did not show an objective response to a doxorubicin containing chemotherapy regimen. This chemotherapy treatment was administered to the patient during clinical care before the extraction of tissue for the establishment of the model. Importantly, when peposertib was added to PLD, tumor growth was significantly blocked (*p* < 0.0001) throughout the treatment duration, in contrast to all control treatments (Figure 6C). This finding underscores that resistance to doxorubicin-containing therapy can eventually be overcome by simultaneous inhibition of DNA-PK. Also, in this study, all treatments exhibited excellent tolerance (Figure 6D).

## 4. Discussion

In the present study, we present compelling evidence that the cytotoxic activity of Topo II inhibitors can be significantly augmented in synovial sarcoma cell lines by the potent and selective DNA-PK inhibitor peposertib. This holds significant importance, considering that advanced or metastatic synovial sarcoma presents a challenging clinical scenario characterized by limited treatment options and suboptimal outcomes [2].

The results observed in vitro using two widely recognized SS18:SSX fusion gene positive synovial sarcoma cell lines indicate not only an additive, but a synergistic anti-tumor activity of the Topo II inhibitors doxorubicin and etoposide when combined with peposertib (Figure 1 and Supplementary Figure S1). Considering possible clinical application, the tumor cell-specific synergistic activity holds significance within the framework of establishing a therapeutic window for potential clinical implementation of this combined treatment strategy. At a mechanistic level, we have shown that the primary cellular outcome for synovial sarcoma cells treated with the combination involves the initiation of apoptosis, likely through a p53-mediated pathway. (Figure 2, Figure 3 and Figure 4). The pivotal tumor suppressor p53 assumes a crucial function in the cellular reaction to DNA damage by triggering cell cycle arrest and/or apoptosis. [19,20,21]. Our NanoString gene expression analysis and Western blotting experiments reveal p53 hyperactivation when doxorubicin and peposertib are administered concurrently, as evidenced by Ser15 phosphorylation of p53 and the increase in mRNA and protein expression of the cyclin-dependent kinase inhibitor p21, a transcriptional target of p53 (Figure 3 and Figure 4). Similar induction of the p53 pathway after the treatment with peposertib and other DNA damaging agent such as irradiation has been reported before [22]. Several other studies showed that DNA DSBs induce phosphorylation of p53 at Ser15 by Ataxia-telangiectasia mutated (ATM) [23,24]. ATM additionally triggers Chk2 activation, leading to the phosphorylation of p53 at Ser20 which, in turn, disrupts MDM2 binding and leads to stabilization of p53 [25]. NHEJ is the primary cellular pathway to repair DNA DSBs induced by Topo II inhibitors. It is noteworthy that it has been previously demonstrated that DNA-PK negatively regulates ATM activity, and thus ATM may become hyperactivated as a compensatory mechanism in the presence of a pharmacological inhibitor of DNA-PK [26]. We hypothesize that ATM hyperactivation (indicated by elevated pChk2 levels) and the subsequent intensified p53 signaling could mechanistically account for the significant induction of apoptosis in the two synovial sarcoma cell lines following the simultaneous administration of doxorubicin and peposertib (Figure 2). This hypothesis is further supported by the results from our gene expression analyses in SYO-1 cells (Figure 3), which indicated significant transcriptional changes related to p53-dependent pathways, DNA damage repair pathways, and cell cycle and proliferation pathways exclusively in the combination treatment groups. In contrast to numerous other types of tumors, synovial sarcomas seem to exhibit a low occurrence of TP53 gene mutations ( < 10%) [21,27,28]. This observation suggests that a strategy of inducing p53-mediated apoptosis through the proposed combination holds merit as a valid approach in treating synovial sarcoma. Although we could not find studies specifically addressing the p53-dependent antitumor activity of a combination therapy involving a Topo II inhibitor and a DNA-PK inhibitor in solid tumor models, our hypothesis is supported by research conducted on acute myeloid leukemia (AML) cells. In their study, Haines et al. demonstrated that peposertib sensitized AML cells with a functional p53 pathway, but not p53-deficient cells, to Topo II inhibitors [22]. Although the study utilized small panels of p53 functional and dysfunctional cell lines instead of isogenic p53-wt/p53-null pairs, distinct responses were observed based on the p53 status. Additional studies with larger cell line panels, including functional characterization of p53-mediated apoptosis induction, are needed to further establish this hypothesis. Crucially, the in vitro results were substantiated by significant anti-tumor effects from the combined treatment approach when applied to mice carrying subcutaneously implanted tumors. (Figure 5 and Figure 6). We were able to establish a well-tolerated treatment regimen for the combined application using liposomal, but also unmodified, doxorubicin (Figure 5A–D). The combination of PLD and peposertib exhibited notably enhanced anti-tumor efficacy compared to unmodified doxorubicin at equivalent doses. This could lead to an improved therapeutic window, a crucial aspect to contemplate if these findings are to be translated into a clinical context. The efficacy studies conducted in two PDX models of synovial sarcoma, which accurately represent human metastatic disease, provide further substantiation for the potential of this combination therapy in human synovial sarcoma. Particularly noteworthy is the PDX model CTG-1173, which originated from a young patient with metastatic disease who showed no response to a clinical regimen containing doxorubicin prior to tissue extraction for model establishment. Our study confirmed this doxorubicin resistance, as evidenced by the lack of significant efficacy of PLD monotherapy at the tested dose. However, the combination with peposertib successfully surmounted the treatment resistance (Figure 6C).

While advancements in genomic profiling have provided insights into potential therapeutic targets in synovial sarcoma, translating these discoveries into effective clinical interventions has proven challenging. The inadequacy of current treatment approaches is highlighted by the limited improvements in overall survival rates with existing therapies over the last decades [1,29]. Innovative approaches that exploit the unique molecular characteristics of synovial sarcoma, such as targeting SS18-SSX fusion gene-related pathways, epigenetic modifications, and immunomodulation, offer a compelling avenue for therapeutic innovation, but it remains elusive if these will demonstrate clinical benefit and result in regulatory approvals for this rare indication [30,31]. Thus, doxorubicin remains an established primary systemic treatment in a palliative context, yielding response rates that span from 16% to 27% along with a median survival of around 18 months from the commencement of first-line systemic therapy [2]. We and others have previously demonstrated that selective pharmacological inhibition of DNA-PK is able to synergistically enhance the anti-tumor activity of anthracyclines, including doxorubicin, in a large variety of cancer cell lines in vitro [14] and xenograft models in vivo. The latter includes breast cancer models [32,33], ovarian cancer models [34,35] and leiomyosarcoma models [36], indications where anthracyclines constitute a crucial component of palliative treatment. Moreover, a clinical phase 1/2 study is currently underway to assess the safety and efficacy of peposertib in conjunction with PLD for patients with ovarian cancer (NCT04092270). Additionally, a recent study has been published with the intention of investigating the combination of PLD with peposertib in patients with leiomyosarcoma (NCT05711615). Our data expands the range of possible tumor indications, implying a scenario where the combination therapy could potentially provide added benefit for patients with synovial sarcoma.

## 5. Conclusions

This study presents compelling evidence that combining Topo II inhibitors with the DNA-PK inhibitor, peposertib, can significantly enhance their effectiveness in well characterized preclinical models of synovial sarcoma. This has important implications for the treatment of advanced or metastatic synovial sarcoma, which currently lacks effective treatment options. 

## Data Availability

The data that support the findings of this study are available from the corresponding author upon reasonable request.

## Supplementary Materials

The following supporting information can be downloaded at: https://www.mdpi.com/article/10.3390/cancers16010189/s1, Figure S1: Peposertib exhibits synergistic antiproliferative activity with etoposide; Figure S2: Peposertib, in combination with doxorubicin, affect cell proliferation and induce apoptosis in synovial sarcoma cell lines; Figure S3: Single agent treatment did not affect gene expression throughout the entire treatment duration; Figure S4: SYO-1 xenografts remain sensitive to PLD + peposertib re-challenge upon relapse; Figure S5: Original, uncropped Western blot membrane; Table S1: Clinical, histological, and molecular data of the PDX models; Table S2: List of antibodies.
