# Supplementary material for: DNA-Dependent Protein Kinase Inhibitor Peposertib Potentiates the Cytotoxicity of Topoisomerase II Inhibitors in Synovial Sarcoma Models
Source: Cancers (Basel). 2023 Dec 30;16(1):189. doi: 10.3390/cancers16010189 (PMC10778103; doi:10.3390/cancers16010189)
Supplement: Supplementary file 1 [file cancers-16-00189-s001.zip › cancers-2768717-Supplementary Figures.pdf]

Supplemental figures

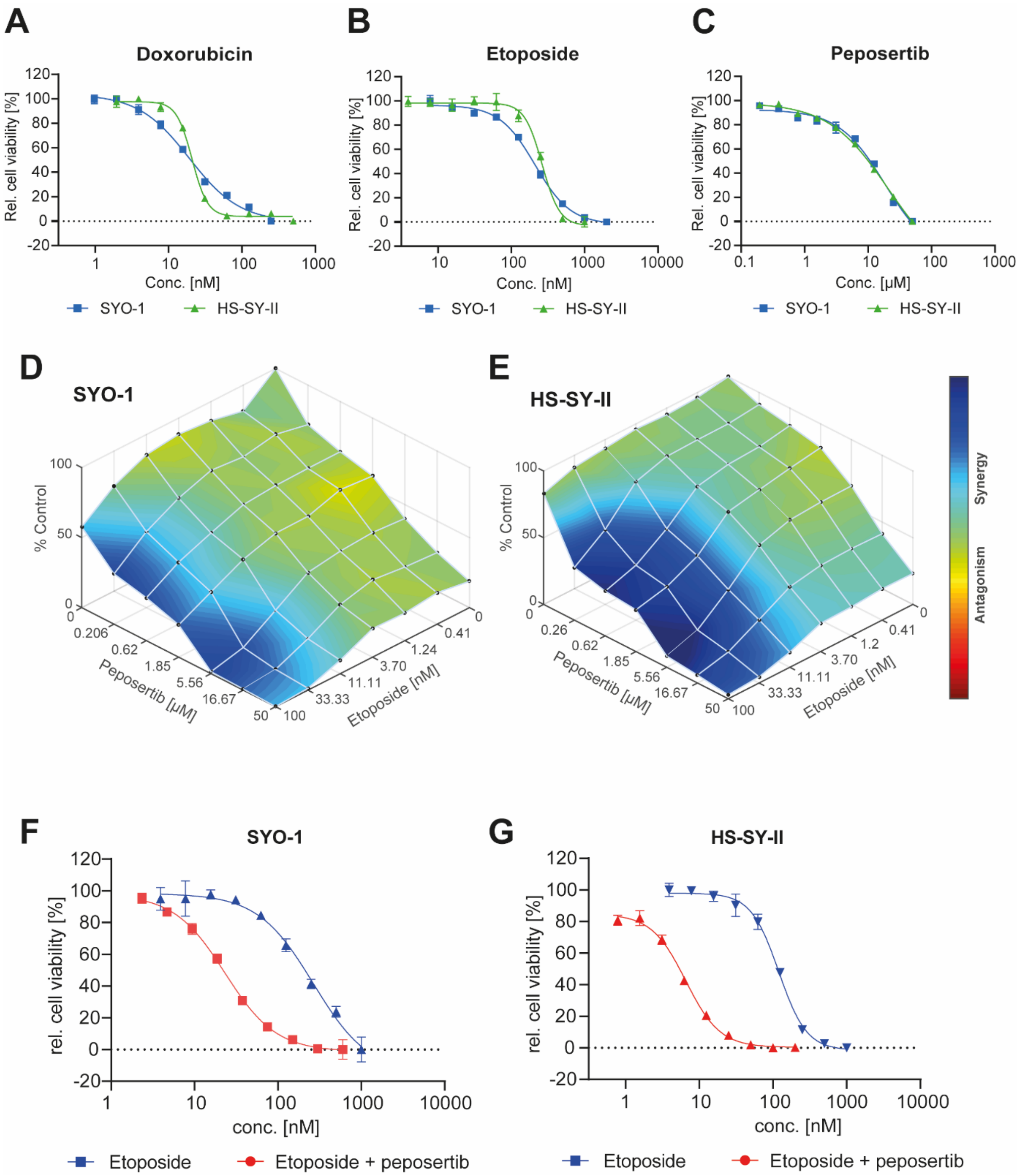

Figure S1. Peposertib exhibits synergistic antiproliferative activity with Etoposide. SYO-1 and HS-SY-II cells cell viability following 168 hours of exposure to increasing concentrations of (A) doxorubicin, (B) etoposide, or (C) peposertib as assessed by Alamar Blue assay. Overlays of Bliss synergy matrices on combination dose response surfaces for SYO-1 (D) and HS-SY-II (E) cells treated with etoposide and peposertib for 168 h. Potentiation of etoposide cytotoxicity by 1 µM peposertib on (F) SYO-1 and (G) HS-SY-II cells as measured using an Alamar Blue viability assay at 168 h post treatment.

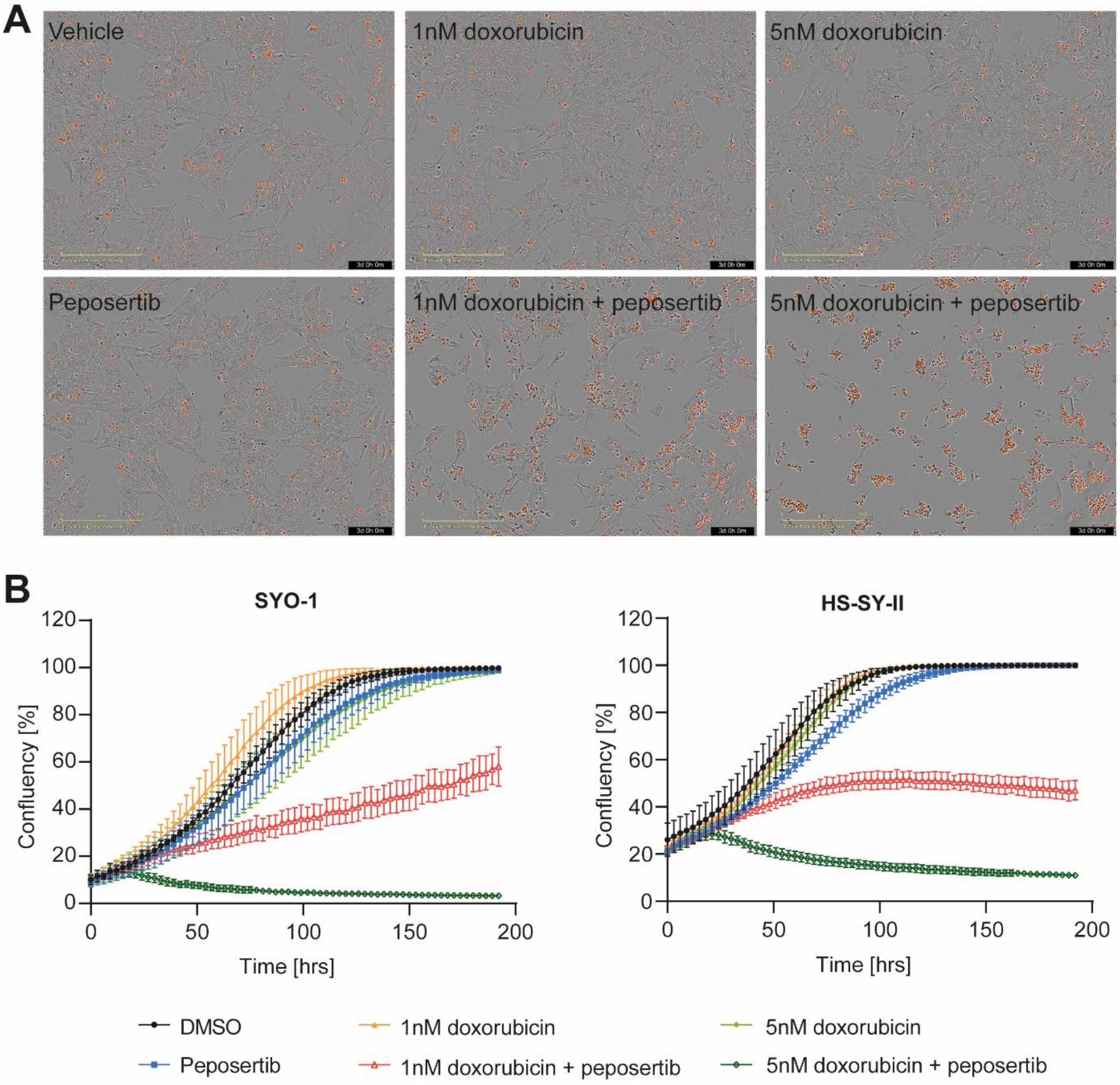

Figure S2. Peposertib, in combination with doxorubicin, affect cell proliferation and induce apoptosis in synovial sarcoma cell lines. (A) Incucyte® bright-field images of HS-SY-II at 72 hours post treatment at 10x magnification overlay with AnnexinV-Red staining. Images are representative from 3 independent replicates. (B) Quantifications of SYO-1 and HS-SY-II confluency over the course of treatment as monitored using the Incucyte®. .

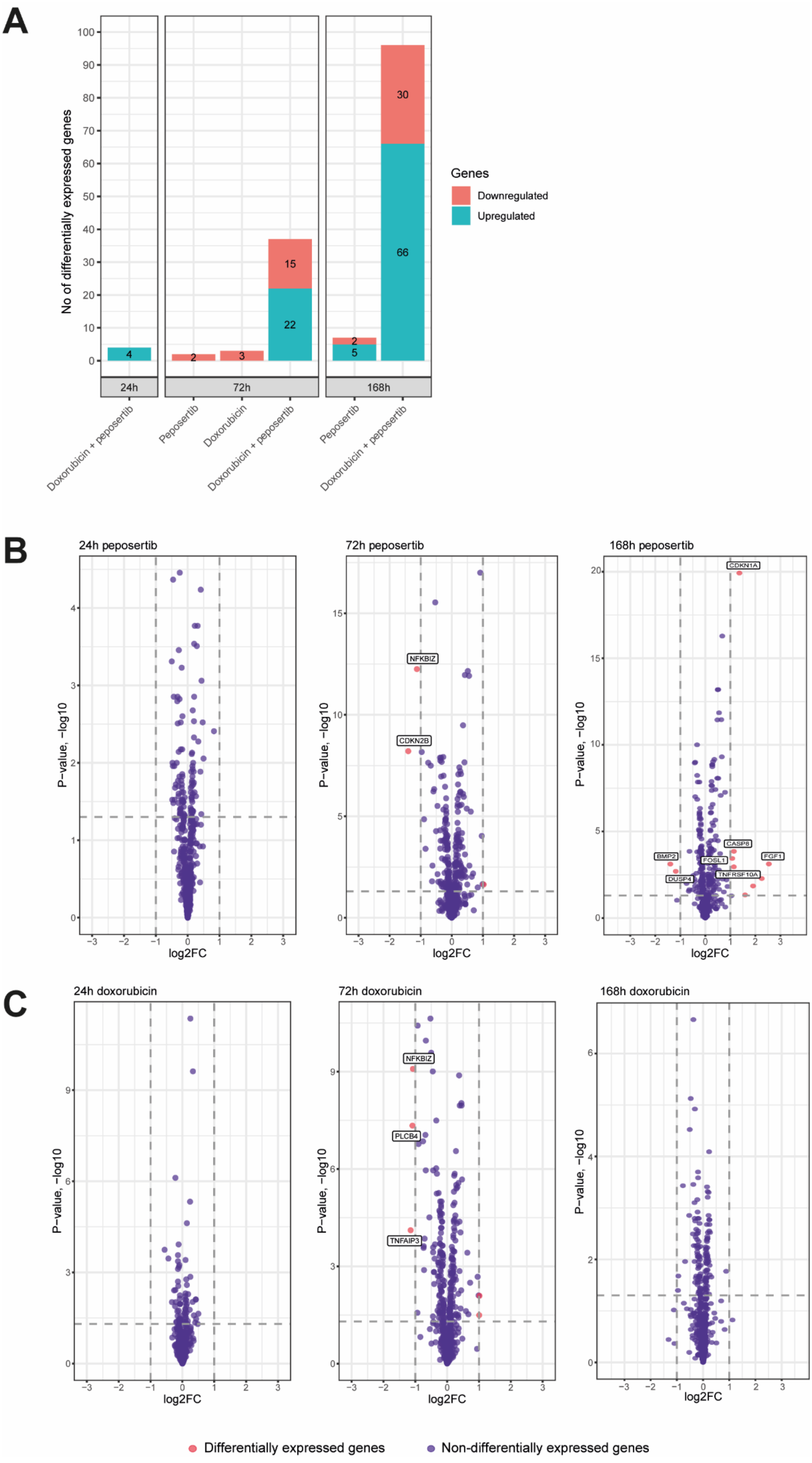

A
No of differentially expressed genes
24h
72h
168h
Doxorubicin + peposertib
Peposertib
Doxorubicin
Doxorubicin + peposertib
Peposertib
Doxorubicin + peposertib
Genes
Downregulated
Upregulated
B
24h peposertib
72h peposertib
168h peposertib
NFKBIZ
CDKN2B
CDKN1A
CASP8
BMP2
FOSL1
FGF1
DUSP4
TNFRSF10A
C
24h doxorubicin
72h doxorubicin
168h doxorubicin
NFKBIZ
PLCB4
TNFAIP3
P-value, −log10
log2FC
Differentially expressed genes
Non-differentially expressed genes

Figure S3. Single agent treatment did not affect gene expression throughout the entire treatment duration. (A) Number of differentially regulated genes upon peposertib, doxorubicin or Peposertib + doxorubicin treatment at 24, 72 and 168 hours. Volcano plots highlighting differentially regulated genes (red dots) in SYO-1 cells identified by limma after exposure to either peposertib (B) or doxorubicin (C) treatment alone at 24, 72 and 168 hours.

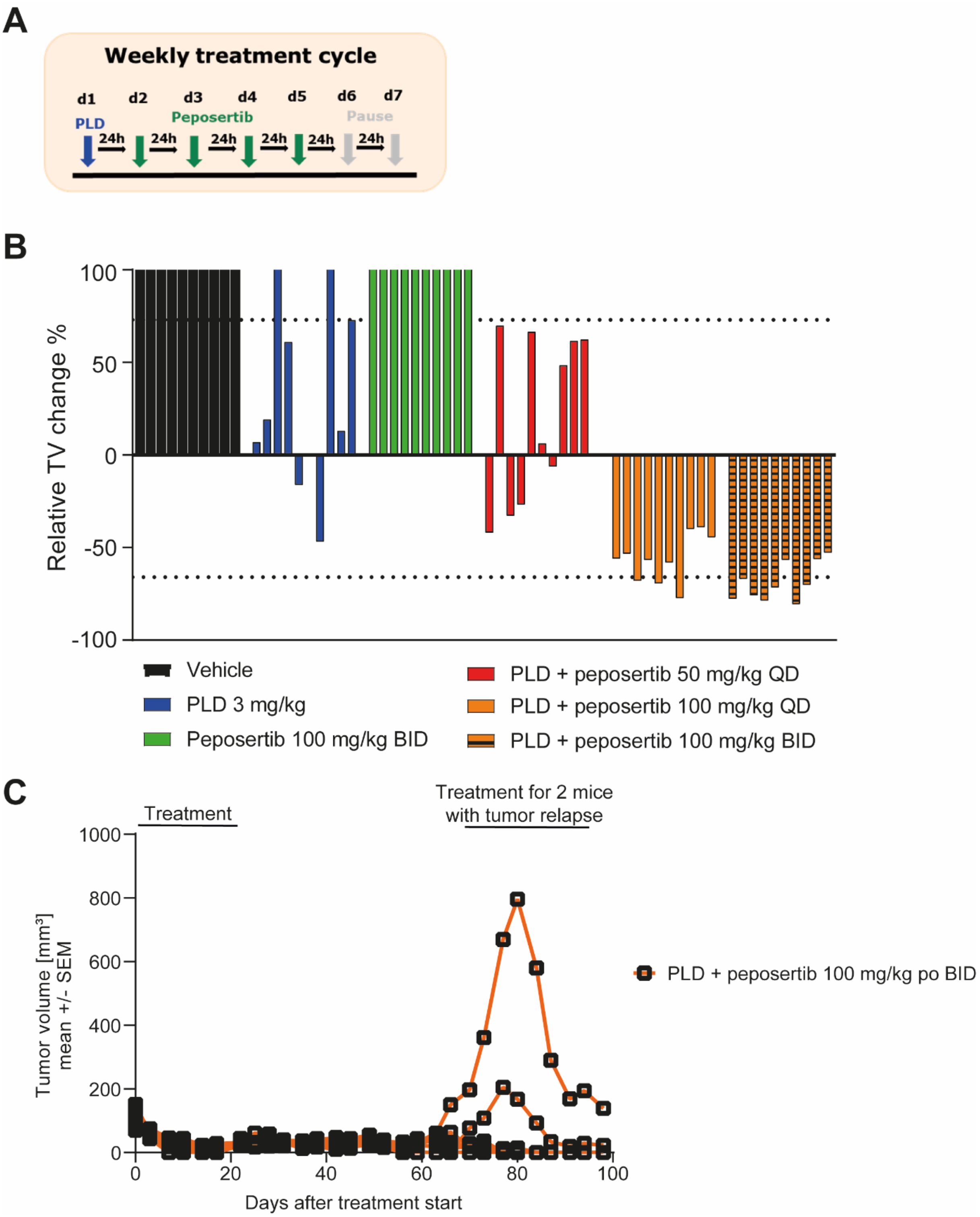

Figure S4. SYO-1 xenografts remain sensitive to PLD + peposertib re-challenge upon relapse. (A) Scheme of the weekly treatment regimen applied in the *in vivo* efficacy studies for doxorubicin/PLD combinations with peposertib. (B) Percentage of changes in tumor size from baseline at day 22 after treatment with vehicle, peposertib, PLD or the combination of varying doses of peposertib with PLD. (C) tumor growth in individual mice treated with PLD and 100 mg/kg BID peposertib. Two tumors relapsed and regressed upon re-challenge with the initial treatment regimen.
