# Supplementary figures and images for: DNA-Dependent Protein Kinase Inhibitor Peposertib Potentiates the Cytotoxicity of Topoisomerase II Inhibitors in Synovial Sarcoma Models
Source: Cancers (Basel). 2023 Dec 30;16(1):189. doi: 10.3390/cancers16010189 (PMC10778103; doi:10.3390/cancers16010189)

SYO-1 cell line

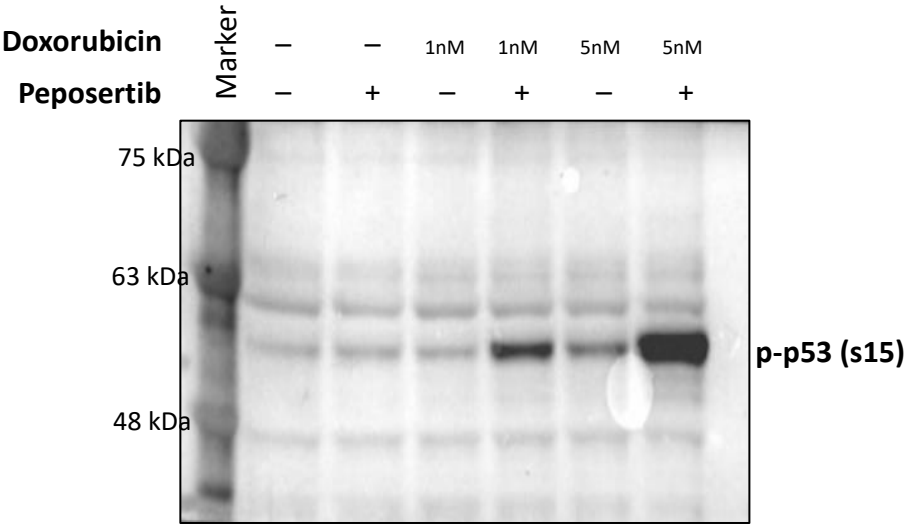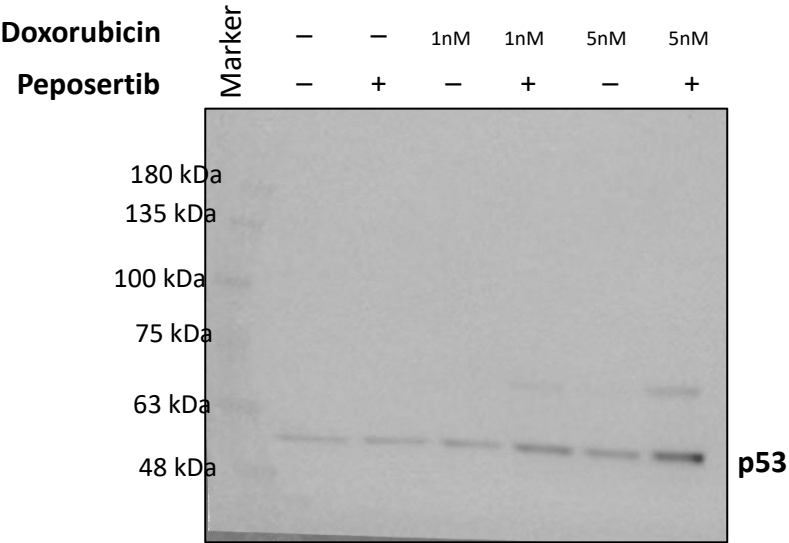

**SYO-1 cell line**

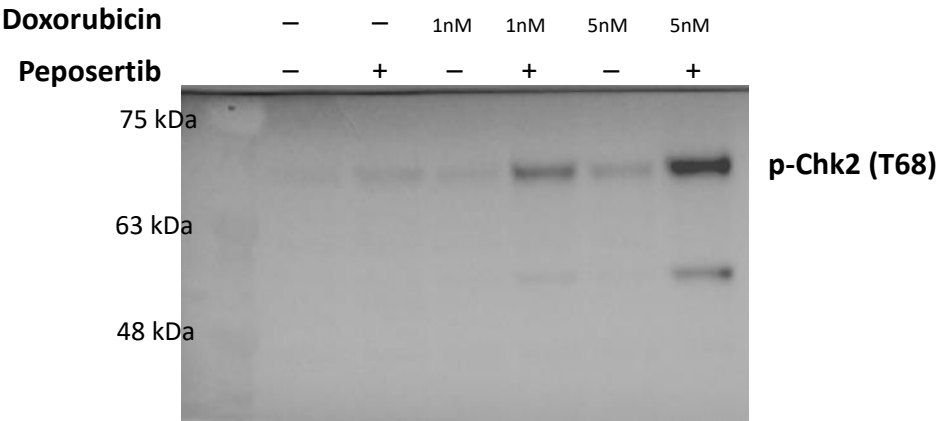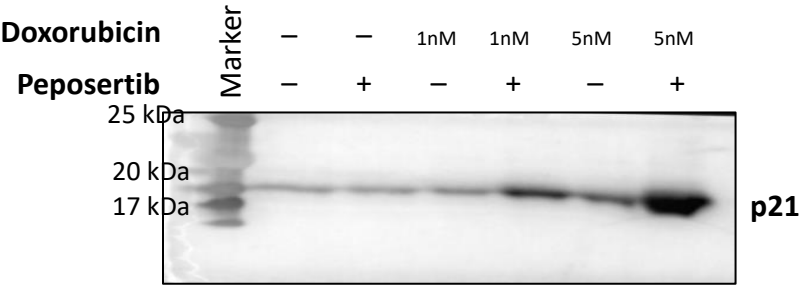

SYO-1 cell line

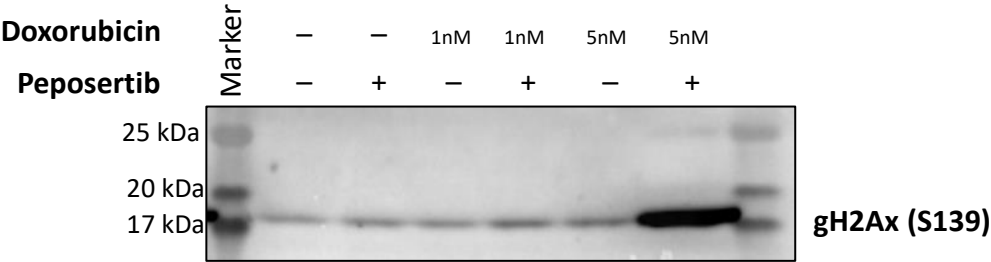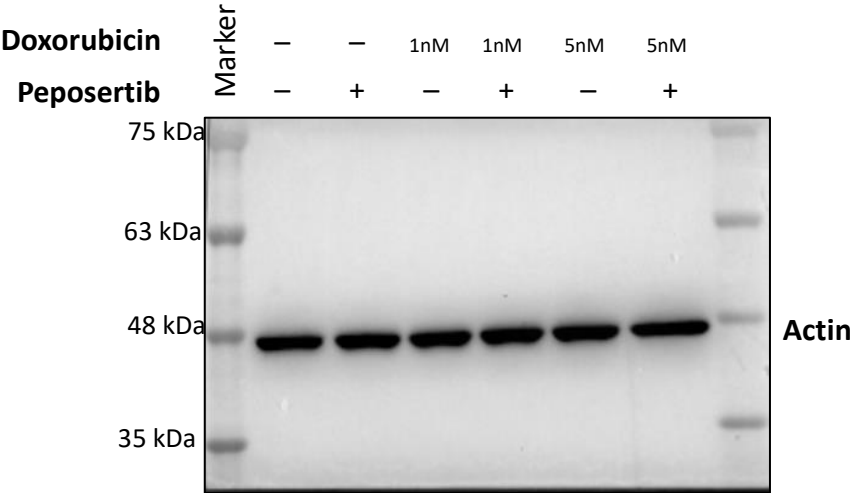

HS-SY-II cell line

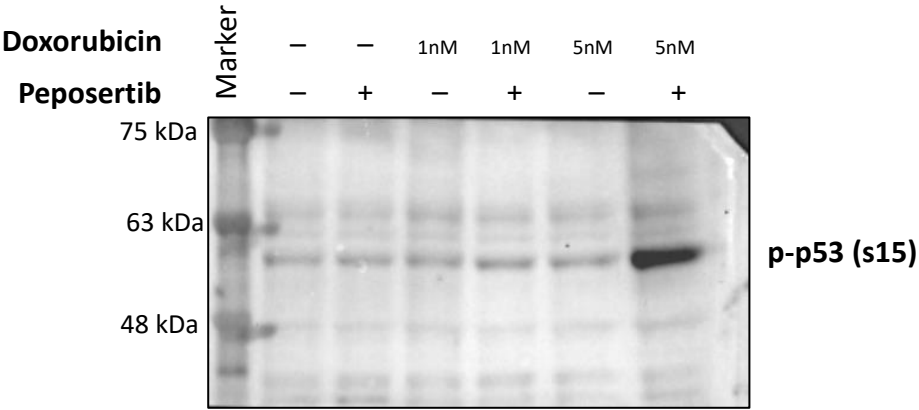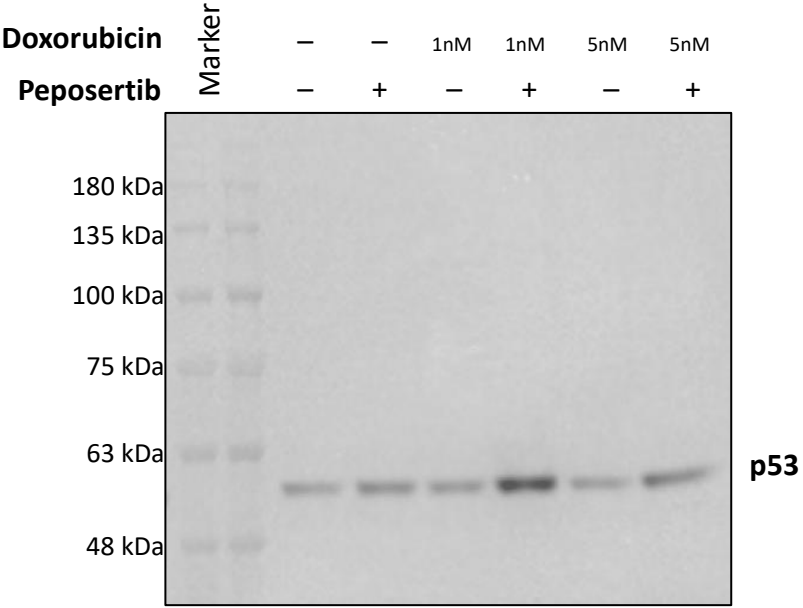

HS-SY-II cell line

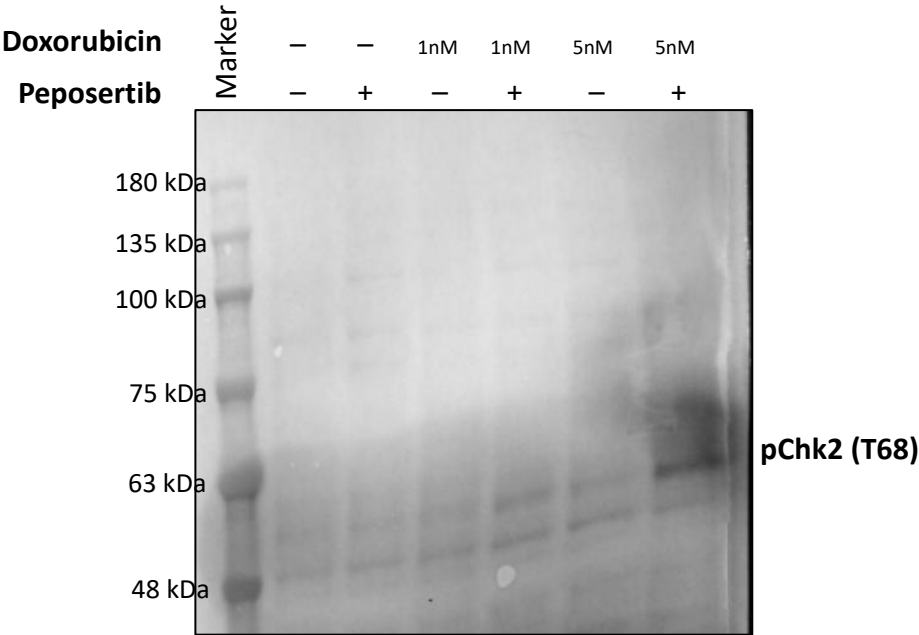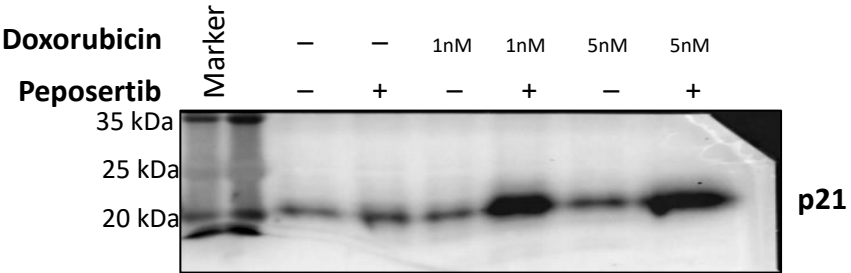

HS-SY-II cell line

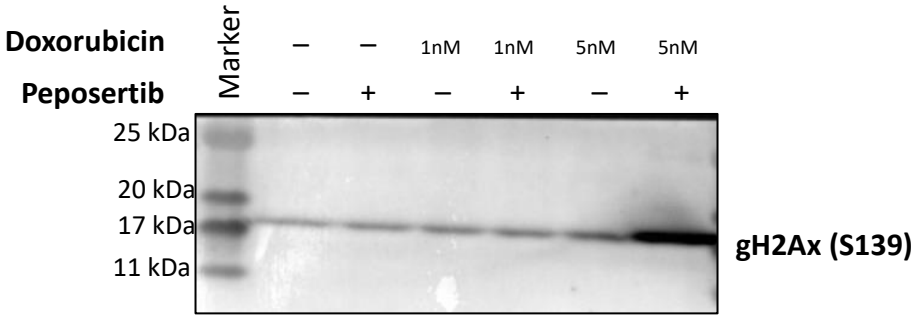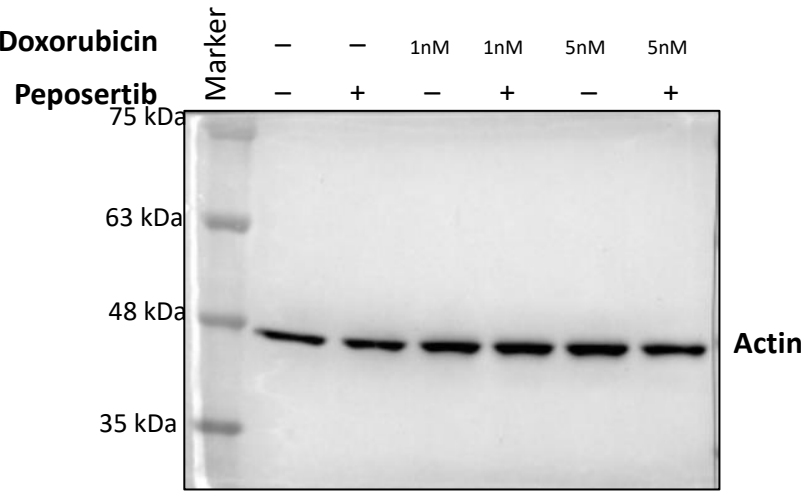

Supplement: Supplementary file 1 [file cancers-16-00189-s001.zip › Figuere S5 Original, uncropped Western blot membrane.pdf]
